# The effects of high-monosaccharide diets on development and biochemical composition of white-eyed mutant strain of house cricket (*Acheta domesticus*)

**DOI:** 10.1038/s41598-021-00393-5

**Published:** 2021-10-27

**Authors:** Jacek Francikowski, Marta Potrzebska, Elżbieta Szulińska, Monika Tarnawska, Zoltan Radai, Bartosz Łozowski, Bartosz Baran, Michał Krzyżowski

**Affiliations:** 1 Institute of Biology, Biotechnology and Environmental Protection, Faculty of Natural Sciences, University of Silesia in Katowice, Bankowa 9 st, 40-007 Katowice, Poland; 2 Lendület Seed Ecology Research Group, Institute of Ecology and Botany, Centre for Ecological Research, Vácrátót, Hungary

**Keywords:** Analytical biochemistry, Biological models, Experimental organisms, Metabolism, Carbohydrates, Biochemistry, Physiology, Developmental biology, Disease model, Experimental organisms, Animal physiology

## Abstract

Tryptophan (TRP) is one of the essential amino acids in the animal body. Its exogenicity and low concentrations mean that it can be regarded as one of the key regulatory molecules at the cellular as well as physiological level. It has been shown to have a number of essential functions, such as in the production of other biologically active molecules. The main objective of this project was to investigate the effects of a high monosaccharide diet (HMD) on a hemimetabolic insect—house cricket (*Acheta domesticus*) and a mutant strain with impaired visual pigment synthesis (closely related to the tryptophan and kynurenine (KYN) metabolic pathway)—white eye. This study was aimed at determining the effects of glucose and fructose on cricket development and biochemical composition. A parallel goal was to compare the response of both cricket strains to HMD. ELISA assays indicated dysfunction of the TRP-KYN pathway in white strain insects and an elevated KYN/TRP ratio. Biochemical analyses demonstrated the effects of HMD mainly on fat and glycogen content. A decrease in food intake was also observed in the groups on HMD. However, no changes in imago body weight and water content were observed. The results of the study indicate a stronger response of the white strain to HMD compared to the wild-type strain. At the same time, a stronger detrimental effect of fructose than of glucose was apparent. Sex was found to be a modulating factor in the response to HMD.

## Introduction

Tryptophan (TRP) is one of the essential amino acids in the animal body. Its exogenicity and low concentrations mean that it can be regarded as one of the key regulatory molecules at the cellular and physiological level^1–3^. It has been shown to have a number of important functions, such as in the production of other biologically active molecules, establishing the interface between the host cells and the gut microbiome, or as a limiting amino acid in protein synthesis^1,4,5^. ABC family transporters (ABCG1/white) are responsible for the uptake of TRP into the cells^6,7^. Approximately 95% of the tryptophan supplied to the body participates in the kynurenine (KYN) pathway (TRP-KYN). The transition from tryptophan to kynurenine is catalysed by tryptophan 2,3 dihydrooxygenase (TDO/vermillion), indole 2,3 dihydrooxygenase (IDO) and kynurenine formamidase (CG9542)^8^. Further reactions produce, among others, kynurenic acid, anthranilic acid and 3-hydroxykynurenine. In further metabolic processes, 3-hydroxyykynurenine is the primary source of NAD in vertebrates and, respectively, of ommochromes in the arthropods^9^. The remaining 5% of tryptophan is metabolised through a pathway initiated by the activity of the enzyme tryptophan monooxygenase, leading to the production of serotonin and melatonin. These regulate processes related to metabolism, sleep and wakefulness, as well as behaviour, development and the functioning of organs^10,11^. As recent studies have shown, TRP and its metabolites may also be considered as agents involved in regulating carbohydrate metabolism^12,13^. Several studies investigating mammals, including humans, with impaired sugar metabolism, such as in type II diabetes (T2D) and insulin resistance (IR), have suggested this conclusion^14,15^. In mammals that exhibit hyperglycemia and its symptoms, elevated blood levels of kynurenine and its derivatives were also observed^16,17^. Studies on the fruit fly *Drosophila melanogaster,* and the nematode *Caenorhabditis elegans* have demonstrated that the carbohydrate metabolism’s regulatory pathways and mechanisms are highly conservative and evolutionary stable; thus, the obtained results could be applicable to mammals to a considerable extent^18,19^. In insects, there are comparatively fewer studies (mainly carried out using the *D. melanogaster* model) showing similar adverse effects of high sugar diet (HSD) or high monosaccharide diet (HMD), leading to IR in the larvae and then T2D in the imago (elevated glucose, trehalose and insulin-like peptides (ILPs) in the haemolymph)^19^. Concurrently, delayed pupal stage attainment, reduced assimilation, metabolic shift to fat accumulation in larvae and bodyweight reduction in imago were also observed^20,21^.

*Drosophila* eye colour mutants are the primary models to study the role of genes and proteins related to tryptophan metabolism in behaviour, physiology and during development^22^. The alteration of eye colour in these strains results from dysfunction of the TRP pathway and disruption of the ommochrome synthesis^23,24^. In this case, *D. melanogaster* mutants are valuable models to study the interaction of disorders of TRP metabolism and the effects of a high-carbohydrate diet on animal development and diabetes physiology. Mutant fly strains such as *white* and *vermilion* are examples of organisms with modified KYN levels due to dysfunctions of the critical enzymes such as TDO or White protein^25^. It is noteworthy that TDO activity and KYN synthesis in insects is primarily related to the fat body, the primary storage site for reserve compounds in insects^26^. Two studies indicate that reduced availability of TRP or the concentration of its metabolites in *D. melanogaster* mitigates the negative effects of HSD^27,28^. In both mutant lines, a reduced effect of HSD on larval developmental duration was observed in comparison with the control wild-type. Hitherto white eye colour mutant was also discovered in the house cricket *Acheta domesticus*, in a laboratory population bred at the Institute of Biology, Biotechnology and Environmental Protection (University of Silesia in Katowice, Poland). Cricket strains bearing mutations affecting the eye colour are characterised by white or yellow eyes. Additionally, the white-eye strain was observed to exhibit dysfunctional tryptophan metabolism and lack of ommochrome pigments^29^. In the available literature, the data about the effects of HSD on hemimetabolous insects like crickets is scarce^30^. Furthermore, there are no data available on the hemimetabolous TRP-KYN pathway mutants strains response to a high-sugar diet. However, the available knowledge about sugar metabolism in hemi- and holometabolous insects suggests substantial evolutionary conservation and physiological similarity^31^. This research's central hypothesis is that white eye mutant strains of crickets are less sensitive to high carbohydrate diets. This study aimed to investigate the effects of high concentrations of monocarbohydrates (glucose, fructose) in food on development, mortality, and biochemical composition in wild-type (black-eyed) and mutant (white-eyed) strain of house cricket, *Acheta domesticus*.

## Results

### Concentration of KYN/TRP and 5-HT in hemolymph

**Figure 1:**
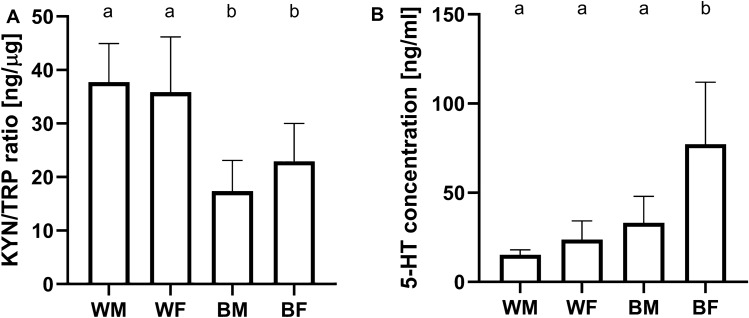
Average values (mean ± SD, n = 10) of (**A**) kynurenine/tryptophan ratio (ANOVA F (3, 36) = 16.23, P < 0.0001) and (**A**) serotonin concentration in hemolymph for imagos (Brown-Forsythe ANOVA 15.77 (3, 11.01) P = 0.0003). Males (M) and females (F) from white (W) and black-eyed (B) strain. Different letters denote groups differing significantly; (**A**) Tukey's multiple comparisons test, P < 0.05 (**B**) Dunnett's T3 multiple comparisons test, P < 0.05.

Measurements of KYN and TRP concentrations in hemolymph revealed differences in KYN/TRP ratio values between groups (P < 0.0001). Elevated about 50% KYN/TRP ratio values were observed in both sexes’ imagoes in the white-eyed strain to wild-type. This indicates a metabolic shift toward KYN in relation to TRP in insects from the white strain. As in the case of white strain insects, there were no differences in values between the sexes in the wild-type (Fig. 1A).

Analysis of serotonin content in the hemolymph of crickets across sexes and strains indicated that there were evident differences within this trait (ANOVA, P = 0.0003). Significantly lower values were observed in females of the white strain in comparison to females of the wild-type. Within the wild type crickets, there were differences in 5-HT concentration between the sexes—females had a higher concentration of 5-HT than males; this difference, however, was not observed in the white strain (Fig. 1B).

### Growth and mortality of larvae

**Figure. 2:**
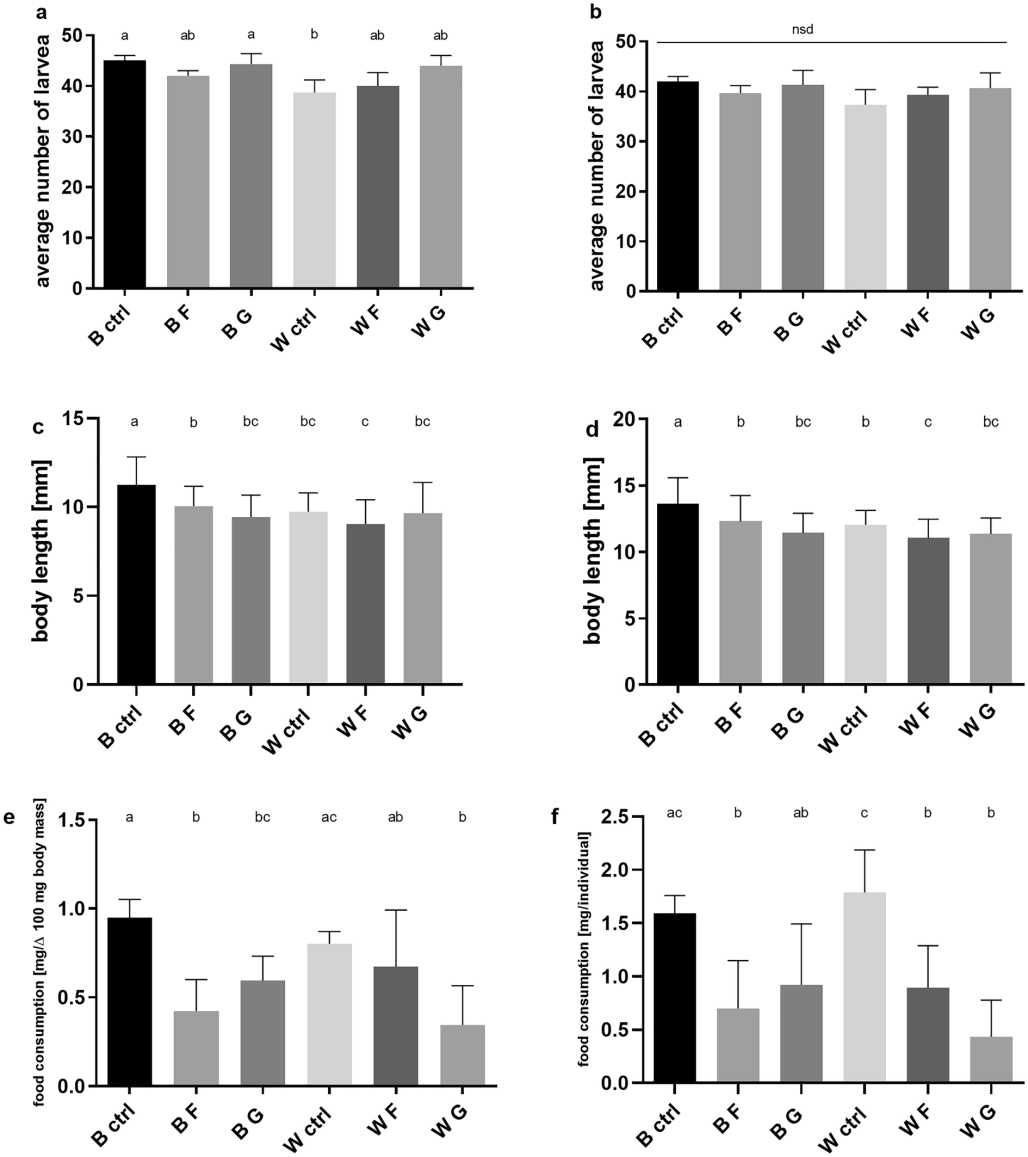
Effects of HMD on larvae mortality, body length and food consumption (*B* black-eyed strain, *W* white-eyed strain, *CTRL* control group, *F* fructose, *G* glucose). Average (mean ± SD, n = 4) larvae number in two time points: (**a**) 30th (ANOVA F (5, 12) = 5.003, P = 0.0105) and (**b**) 40th day after hatching (ANOVA F (5, 12) = 1.529, P = 0.2529). Tukey's multiple comparisons test, P < 0.05. Average larvae body length (mean ± SD, n = 4) in two time points: (**c**) 30th (Brown-Forsythe ANOVA test, F = 13.67 (5, 234.9), P < 0.0001) and (**d**) 40th day (Brown-Forsythe ANOVA test, F = 16.33 (5, 222.0), P < 0.0001) after hatching. Dunnett's T3 multiple comparisons test, P < 0.05. Average (mean ± SD, n = 6) food consumption expressed as (**e**) amount of eaten food per 100 mg of increased body mass (ANOVA F (5, 30) = 8.575, P < 0.0001) by crickets larvae between 30–35 day of development and (**f**) the amount of eaten food per individual (ANOVA F (5, 30) = 10.07, P < 0.0001). Different letters denote groups differing significantly. Tukey's multiple comparisons test, P < 0.05.

Analysis of the mortality of larvae over time failed to show any discernible changes by strain or diet. The only observable difference was reduced abundance in the white strain control group (compared to the wild-type control group and glucose-treated group at day 30) (Fig. 2a). On the 40th day, all the numerosity differences between groups have vanished, as confirmed by statistical indicators of homogeneity (Fig. 2b).

Significant differences in the body length of larvae on the 30th day can be observed between the control group of the wild-type crickets and the other groups. The mean body length of these groups is significantly lower. Simultaneously, the lower mean body length of the larvae of the white strain fructose group compared to the fructose group of the wild type is discernible (Fig. 2c). On the 40th day, the differences become more prominent, and significant differences emerge between the treatment groups: the white strain control group and the fructose group of wild type (still, all groups differ from the wild type control group) (Fig. 2d).

### Larvae food consumption

Conducted food consumption test show statistically significant differences between groups. No differences were observed between the control groups of wild-type and white strains. Food consumption measured as the amount of food per individual indicated differences between the wild-type and the fructose treated group with the lower value in the latter. In the white strain, there was a difference between the control and the two HMD groups—consumption was lower in these groups (Fig. 2e). When the consumption was re-calculated as food intake per 100 mg of body mass increase (food mg/∆100 mg of body mass), the wild-type groups’ average relationships remained the same. In the white strain groups, a statistically significant difference was apparent only in comparison between the control group and the glucose treated group (Fig. 2f). There was a decrease in consumption in the monosaccharide treated groups compared to the control group.

### Larvae frass caloricity

**Figure 3:**
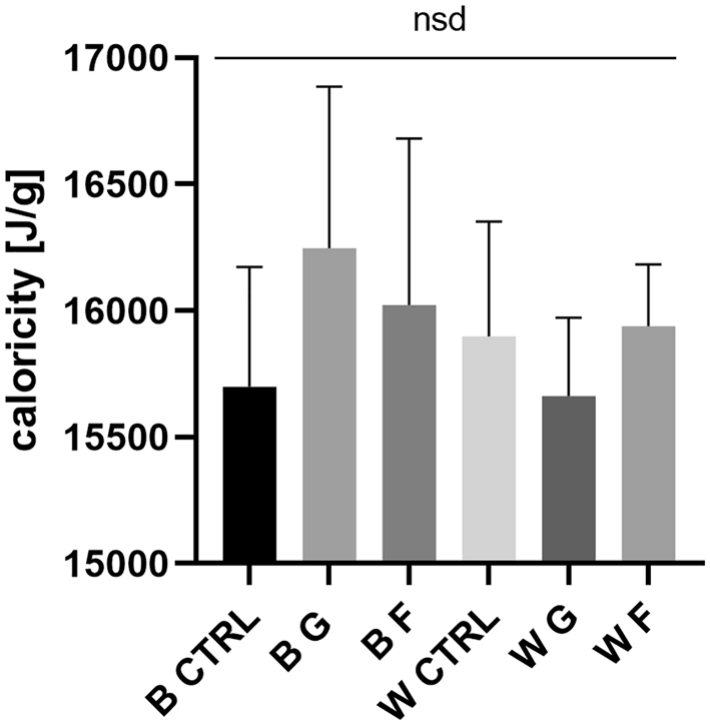
Wild-type and white strain larvae frass caloricity (mean ± SD, n = 4) (*B* black-eyed strain, *W* white-eyed strain, *CTRL* control group, *F* fructose, *G* glucose). One-way ANOVA F (5, 19) = 0.8612, P = 0.5247. *Nsd* no statistical difference.

Statistical analysis of the caloric content of larval frass showed no differences between groups concerning both lines and used monosaccharides (ANOVA P = 0.5247) (Fig. 3).

### Effect of monosaccharides on maturing success

**Figure 4:**
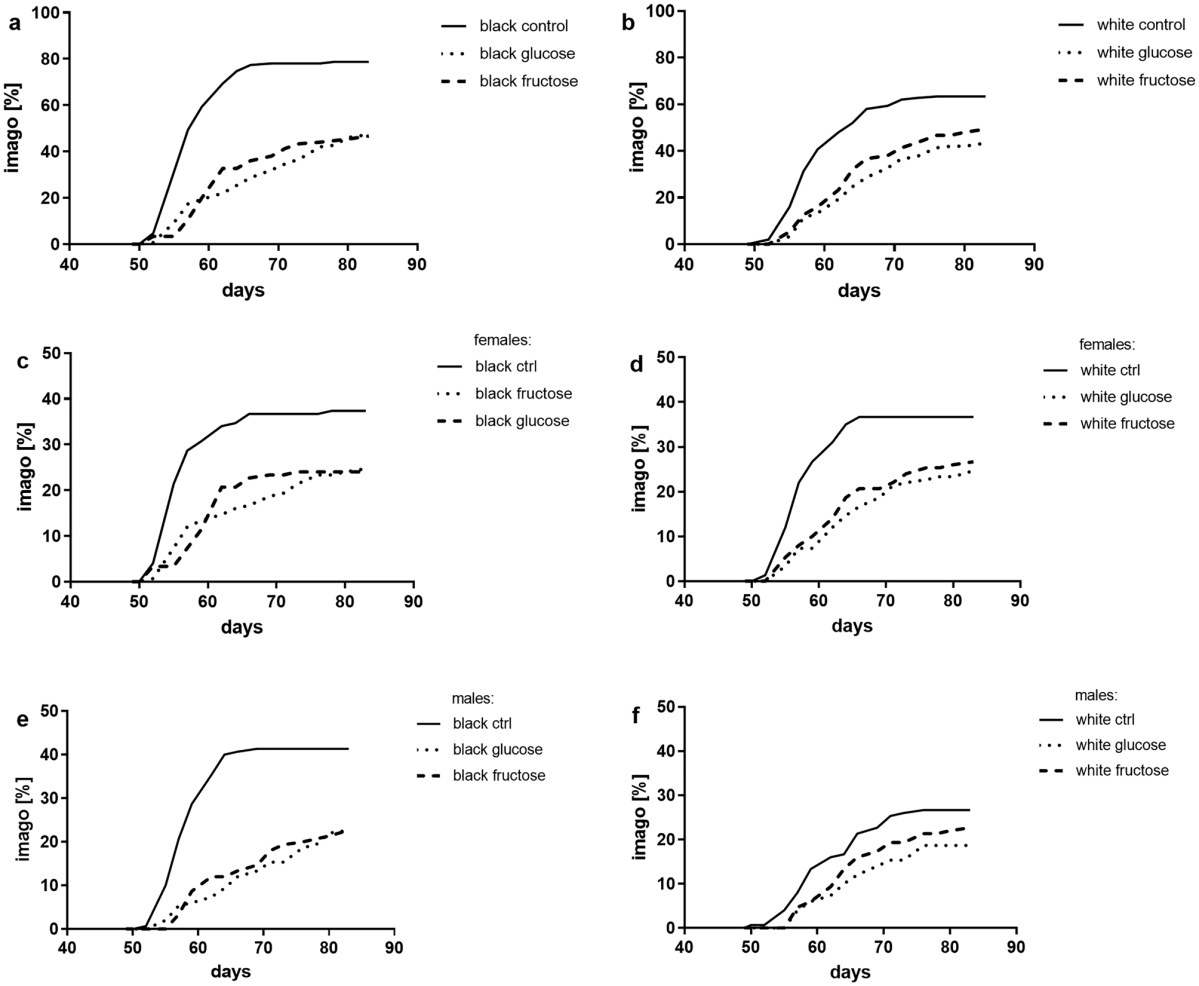
The success of imago stage reaching presented as cumulative percentage curves: (**a**) black-eyed strain, (**b**) white-eyed strain, (**c**) black-eyed strain females, (**d**) white-eyed females, (**e**) black-eyed strain males, (**f**) white-eyed males.

There is a significant effect of administered monosaccharides on the success of larvae in reaching the imago stage. In general, white strain insects from the control group showed a lower success in reaching the imago (63%) than the wild type (78%). The reduced success of reaching imago in MHD groups in both strains indicates a strong effect of monosaccharides compared to the control groups. Although, the differences in white strain are much less pronounced. When stratified by sex, all HMD groups display an increase in the time required to enter the imago stage and a reduced number of insects reaching the imago stage for both glucose and fructose groups. Both diets show a similar intensity of the effect. Among all the groups, white strain males stand out—the lowest difference is observed between the HMD groups and the control (Fig. 4).

In general males matured in significantly lower proportions than females (z = − 4.41, P < 0.001), and eye color mutants also showed decreased maturing success (z = − 3.63, P < 0.001). Both treatments with fructose (z = − 10.38, P < 0.001) and glucose (z = − 10.25, P < 0.001) decreased maturing success in wild-type and mutant crickets as well, although this effect was milder in eye color mutants in the case of fructose treatment (z = 2.39, P = 0.017), but not in the case of glucose treatment (z = 1.59, P = 0.113). The interactions between sex and phenotype, and sex and treatment were not significant (P > 0.1 for both).

### Effect of monosaccharides on levels of biomolecules: larvae

**Figure 5:**
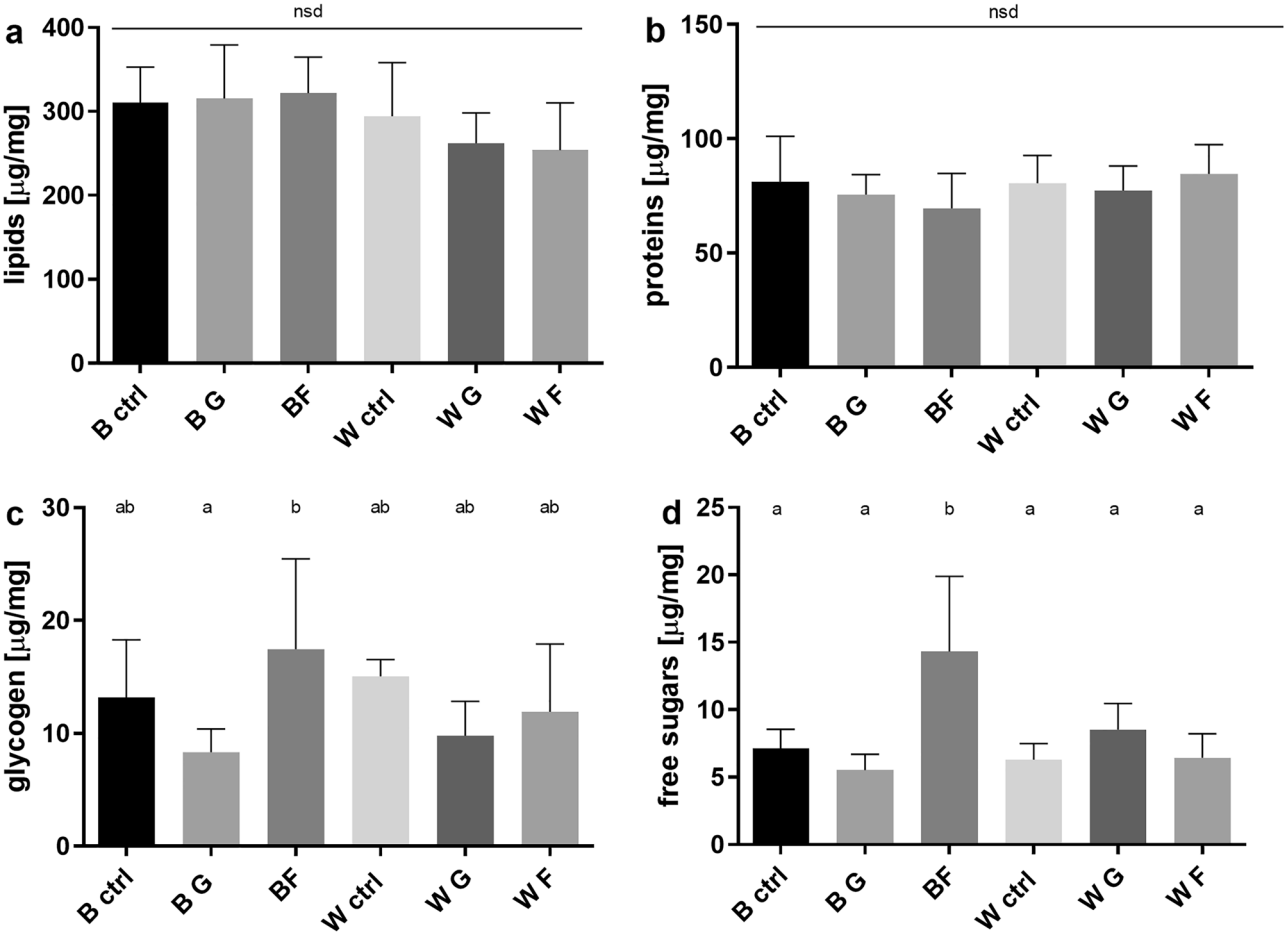
Average concentration (mean ± SD, n = 6) of four major groups of biomolecules in the body of 30-day-old larvae (**a**) lipids (ANOVA F (5, 30) = 1.832,P = 0.1367); (**b**) proteins (ANOVA F (5, 30) = 0.8768,P = 0.5084); (**c**) glycogen (ANOVA F (5, 30) = 2.868, P = 0.0312); (**d**) free sugars (ANOVA F (5, 30) = 8.868, P < 0.0001). *B* black eyed strain, *W* white-eyed strain, *CTRL* control group, *F* fructose, *G* glucose. Different letters denote groups differing significantly; Tukey's multiple comparisons test, P < 0.05.

Comparative analysis showed no differences between the groups regarding protein and fat content at the larval stage (Fig. 5a,b). The difference is apparent for free sugars and glycogen content, where the fructose group of the wild strain displayed the most prominent differences. It displayed higher concentrations of both forms of carbohydrates. In the case of glycogen, this group differs significantly from the glucose group of the wild-type, and the level of free sugars in this group is significantly higher compared to all of the groups (Fig. 5c,d).

In the model testing the effect of sugar treatments on body mass of larvae the interaction term was not significant (F30 = 1.40, P = 0.263), and eye color mutant larvae did not differ in their body mass from wild-type larvae (t32 = − 0.80, P = 0.428). Treatment with fructose had a significant negative effect on body mass (t32 = − 2.16, P = 0.039), while the effect of glucose treatment was not significant (t32 = − 1.36, P = 0.185).

Interaction of treatment and phenotype was not significant in the model on lipids of larvae either (F30 = 0.78, P = 0.467), and neither treatment with glycogen (t32 = − 0.69, P = 0.496) nor with glucose (t32 = − 0.64, P = 0.527) had a significant effect on lipid levels, but mutant crickets tended to have lower lipid levels compared to the wild-type crickets (t32 = − 2.67, P = 0.012).

In the protein level model of larvae the interaction between treatment and phenotype was not significant (F30 = 1.13, P = 0.337). Also, mutant and wild-type crickets did not differ in their protein levels (t32 = 1.17, P = 0.251), and neither fructose (t32 = − 0.68, P = 0.501) nor glucose (t32 = − 0.80, P = 0.430) treatment affected protein content.

Interaction term in the model on glycogen level of larvae was not significant (F30 = 2.20, P = 0.129). Glycogen levels did not significantly vary with phenotype (t32 = − 0.44, P = 0.662) and were unaffected by fructose treatment (t32 = 0.28, P = 0.785), but showed a significant decrease in response to treatment with glucose (t32 = − 2.46, P = 0.020).

In the case of glucose levels, fructose treatment had a significantly positive effect in wild-type crickets (t30 = 4.68, P < 0.001). The effect of fructose treatment in eye colour mutants significantly differed from the effect on wild-type crickets (t30 = − 3.24, P = 0.003), meaning that while fructose treatment increased glucose levels in wild-type crickets, it did not induce change in glucose levels in mutants. Also, while treatment with glucose didn’t have a significant effect on glucose levels in wild-type crickets (t30 = − 1.04, P = 0.309), it caused a marginally significant increase in glucose levels of mutant crickets (t30 = 1.75, P = 0.090).

### Effect of monosaccharides on levels of biomolecules: imago

**Figure 6:**
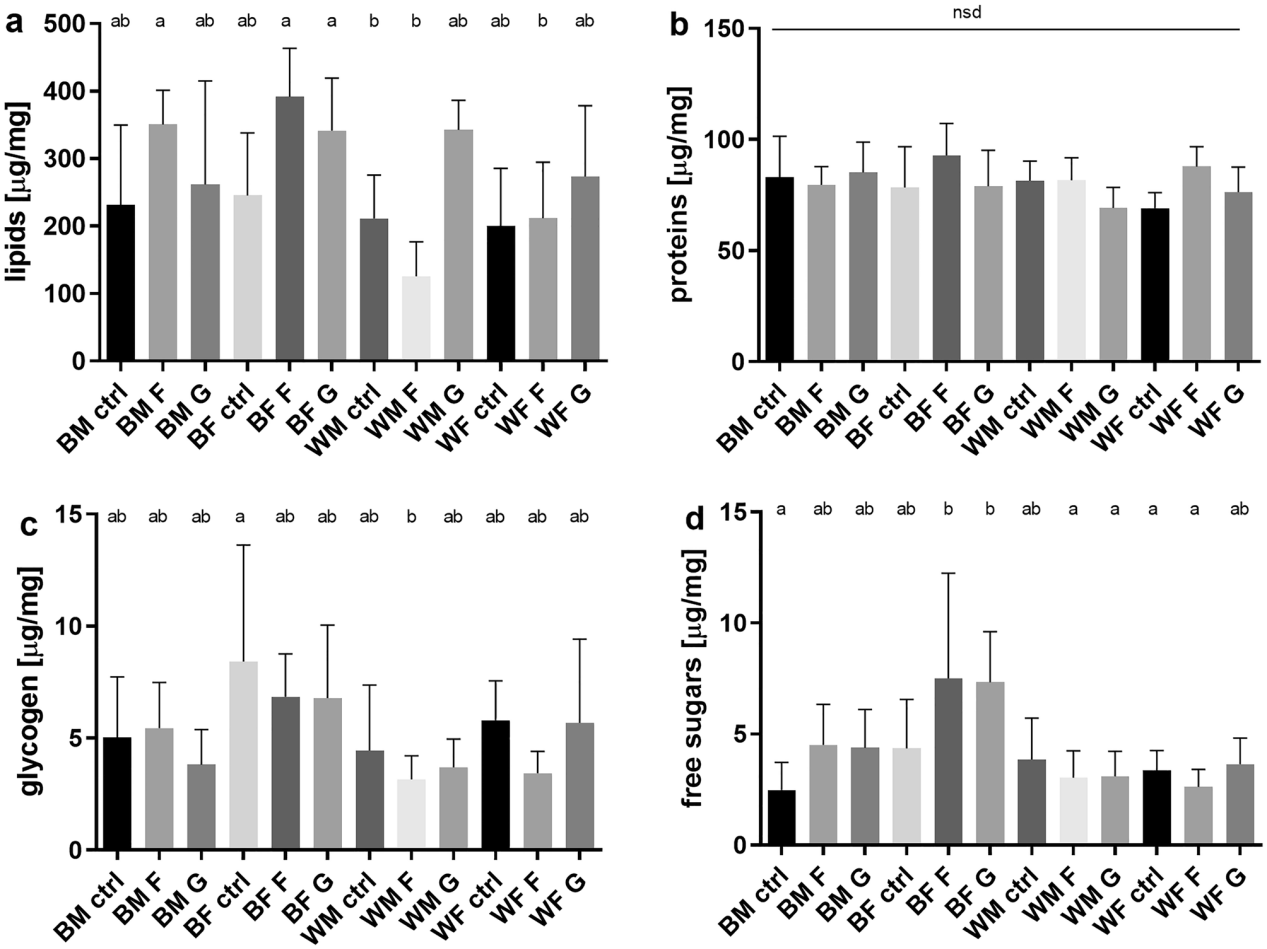
Average concentration (mean ± SD, n = 6) of four major groups of biomolecules in the body of imago (sex divided) (**a**) lipids (ANOVA F (11, 60) = 4.627, P < 0.0001) (**b**) proteins (ANOVA F (11, 60) = 1.767, P = 0.0804) (**c**) glycogen (ANOVA F (11, 60) = 2.182, P = 0.0274) (**d**) free sugars (ANOVA F (11, 60) = 3.989, P = 0.0002). *B* black eyed strain, *W* white-eyed strain, *M* males, *F* females, *CTRL* control group, *F* fructose, *G* glucose. Different letters denote groups differing significantly; Tukey's multiple comparisons test, P < 0.05.

In imago, differences in biochemical composition were observed for all biomolecules except proteins. For lipids, no differences were observed between HMD groups and control groups regardless of sex. However, an increase in lipid concentrations was observed between the sexes of both strains. Both males and females of the wild-type of the fructose group showed elevated lipid concentrations in comparison to males and females of the fructose group from the white strain, respectively (Fig. 6a,b). Generally, no changes in glycogen levels were observed; the only difference was visible between wild-type control females and white males on a fructose diet (Fig. 6c). Whereas distinct differences in free sugar concentrations appeared between the females of the wild-type and white strains. Again, in the wild-type insects wild-type, an increase in concentration is observed compared to the white strain (Fig. 6d).

In the model on adult body mass none of the interaction terms were significant (treatment-phenotype: F62 = 1.29, P = 0.282; treatment-sex: F62 = 1.64, P = 0.202; phenotype-sex: F62 = 2.71, P = 0.105). Eye color mutants were marginally significantly larger than wild type crickets (t67 = 1.69, P = 0.096), and males were generally smaller than females (t67 = − 2.67, P = 0.009). Treatment with fructose (t67 = − 0.04, P = 0.968) or glucose (t67 = 0, P = 1) did not have any significant effects on body mass of adults.

In the model on lipid levels of adults the interaction between treatment and phenotype was significant (F62 = 9.23, P < 0.001), but interaction terms between sex and treatment (F62 = 0.92, P = 0.403), and between sex and phenotype (F62 = 1.05, P = 0.311) were not. In wild type adults fructose treatment had significant positive effect on lipid levels (t67 = 3.64, P < 0.001). The effect of fructose treatment differed significantly in mutant crickets (t67 = − 3.29, P = 0.001), meaning that lipid levels were not affected by fructose treatment in mutant crickets. Additionally, glucose treatment had a marginally significant positive effect on adult lipid levels in both wild type and eye color mutant crickets (t67 = 1.73, P = 0.088).

The interaction term between treatment and sex was marginally significant (F62 = 3.05, P = 0.054) in the model on protein levels of adults, while interactions between treatment and phenotypes (F62 = 0.58, P = 0.566), and between sex and phenotype (F62 = 0.01, P = 0.931) were not significant. Eye color mutant adults had marginally significantly lower protein levels in comparison to wild type adults (t65 = − 1.82, P = 0.073). Treatment with fructose significantly increased protein levels in both phenotypes (t65 = 3.24, P = 0.001), but only in females, as suggests that in males the effect of fructose treatment was significantly lower than in females (t65 = − 2.5, P = 0.015). Glucose treatment did not affect protein levels in adults (t65 = 0.75, P = 0.455).

In the adult glycogen level model none of the interaction terms were significant (treatment-phenotype: F62 = 1.10, P = 0.341; treatment-sex: F62 = 0.74, P = 0.482; phenotype-sex: F62 = 1.26, P = 0.266). Eye color mutant adults had lower levels of glycogen than wild type adults (t67 = − 2.74, P = 0.008). Also, on average males showed lower levels of glycogen in comparison with females (t67 = − 3.07, P = 0.003). Treatment of fructose (t67 = − 1.60, P = 0.115) and glucose (t67 = − 1.23, P = 0.224) did not affect glycogen levels.

Interaction of sex with treatment was not significant in the adult glucose levels model (F62 = 0.42, P = 0.656). However, the interaction between treatment and phenotype (F62 = 4.75, P = 0.012), and also between phenotype and sex (F62 = 8.35, P = 0.005) were significant. The effect of fructose treatment on adult glucose levels was significantly positive in wild type crickets (t64 = 3.20, P = 0.002) but not in eye colour mutants. Similar was the case for glucose treatment, as it only increased systemic glucose levels in wild-type adults (t64 = 3.02, P = 0.004), but not in the mutant strain. Additionally, in wild-type males, there were significantly lower glucose levels compared to females (t64 = − 3.94, P < 0.001), but this sex-based discrepancy was not apparent in eye colour mutant adults. In the control group, there was no difference in glucose levels between wild-type and eye colour mutant adults (t64 = − 1.26, P = 0.212).

### Effect of monosaccharides on imago body mass and water content

**Figure 7:**
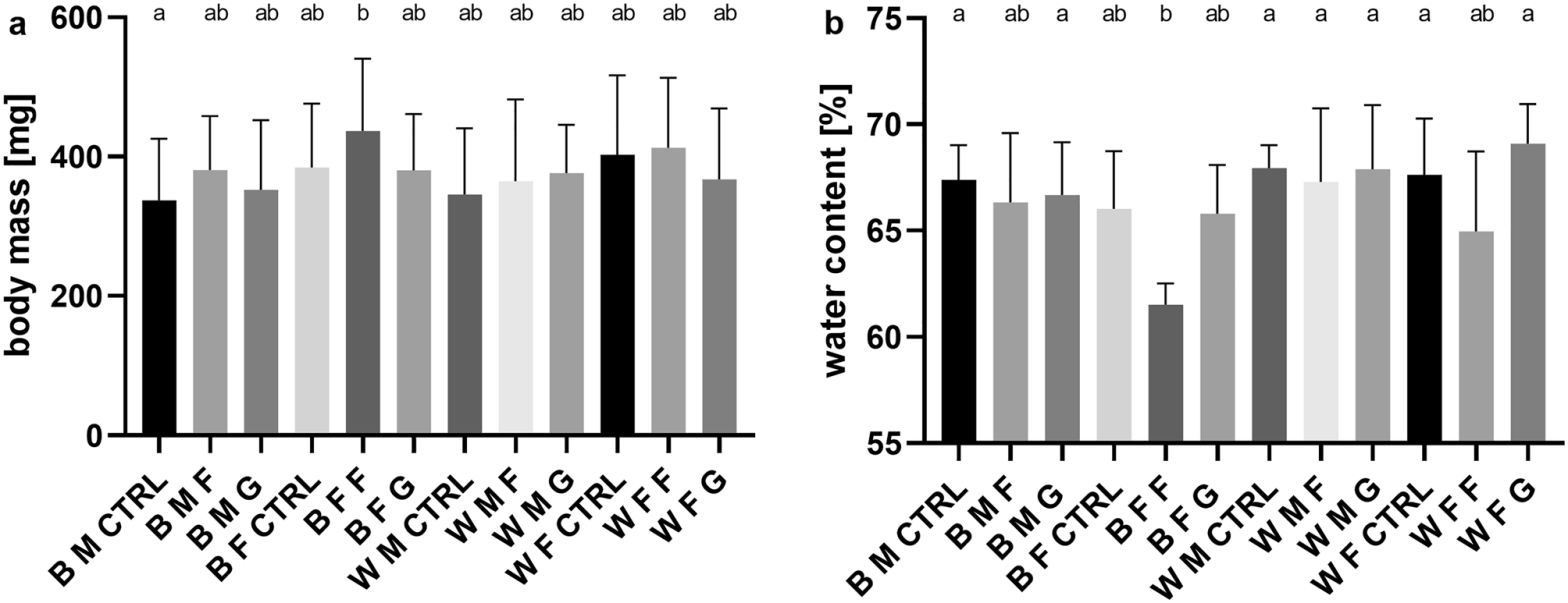
Effects of HMD on imagoes body mass and water content (mean ± SD). (**a**) Imago body mass (fresh body weight) for both sex and strains after HMD treatment (minimum n = 10). One-way ANOVA F (11, 247) = 2.195, P = 0.0153. (**b**) Water content (percentage of fresh body mass) for imagos of both sex and strains after HMD treatment (n = 6). One-way ANOVA F (11, 60) = 3.397, P = 0.0011. *B* black-eyed strain, *W* white-eyed strain, *M* males, *F* females, *CTRL* control group, *F* fructose, *G* glucose. Different letters denote groups differing significantly, Tukey's multiple comparisons test, P < 0.05.

Analysis with one-way ANOVA revealed a difference between groups in body mass (P = 0.0153). A thorough analysis with multiple comparisons showed differences only between wild-type control group males and wild-type fructose group females (Fig. 7a).

Water body content the majority of groups oscillated around 67%. Analysis of body water content using one-way ANOVA revealed a between-group difference (P = 0.0011). The only group distinguished from the others was black-eyed females treated with fructose. Similar, the average value for white-eyed females treated with fructose was lower, but it is not statistically different from other white strain groups (Fig. 7b).

## Discussion

Tryptophan and its metabolites are known to be involved in insect development and metamorphosis. Numerous studies indicate cyclic changes of TRP and KYN concentrations in hemolymph during the moulting stages of insects and during the transition to the imago stage^32,33^. There are many strains of fruit fly with mutations in the TRP-KYN pathway affecting the concentrations of these compounds. The two most common mutations used in HSD reaserch, white and vermillion, lead to decreased tryptophan or kynurenine concentrations in hemolymph^27,28^. Enzymatically regulated KYN formation from TRP begins at the end of the third larval instar in the cells of the anterior region of the fat body^34^. An additional consequence of these mutations is the disruption of the synthesis of ommochromes pigments and the changes of the eye colour phenotype. Apart from *D. melanogaster*, there are also examples of members of other holometabolous orders in which mutation of this pathway has been described^6,35,36^. Sparse reports are available for insects of the hemimetabola group^37,38^. Concurrently, human, rodent and fruit fly studies indicate that TRP-KYN pathway, lateral products and associated perturbations are also related to carbohydrate homeostasis mechanisms^15^. The connection of this pathway with insulin resistance and diabetes is also indicated^39^. Nonetheless, to our best knowledge, there is no information about the effects of the aforementioned mutations in hemimetabolous insects on TRP, KYN, and 5-HT levels or metabolism. The project aimed to investigate the interactions of the TRP-KYN pathway with HMD and its influence on the physiology and development of the hemimetabolous insect; house cricket (*Acheta domesticus*), using a white-eyed mutant strain.

The first stage of the presented research was to check whether white-eyed cricket mutant strain, in addition to their altered eye pigmentation, also exhibit alterations in the TRP-KYN pathway. Colourimetric ELISA assay revealed differences in KYN/TRP ratio and serotonin levels in the hemolymph of white strain crickets compared to the wild-type (Fig. 1). This supports the hypothesis that the TRP-KYN pathway is disrupted in white eye crickets. The effect of the mutation is associated with an increase in the KYN/TRP ratio in both sexes of the white strain and a decrease in 5-HT in white strain females (Fig. 1). The increase in KYN/TRP ratio indicates an accumulation of kynurenine in the hemolymph, which may be the result of blocking the ommochromes synthesis pathway^40^. This causes the white eye cricket strain to more closely resemble a *D. melanogaster* cinnabar mutant strain than a white or vermilion^41^. It is hardly possible to address the results on TRP and KYN concentrations in the body of fruit flies as they were determined in the heads and not in the haemolymph**.** Only studies on stick insects report TRP concentrations in haemolymph oscillating in a similar range to that observed in cricket^33^. As shown in studies involving locusts and flies, the artificial elevation of TRP, KYN, or other metabolites participating in this pathway is toxic or lethal and has been shown to produce cytotoxic and neurotoxic effects^42,43^. This can also affect insect development and survival, as seen in white-eyed crickets, characterised by slower growth in juvenile body length, lower success in reaching the imago stage, and longer mean developmental time than wild-type insects (Fig. 4). This is a probable consequence of elevated KYN concentrations and decreased 5-HT concentrations. To the authors’ knowledge, there are no reports of changes in growth rate, dynamics or effectivity of imago attainment in cinnabar mutants (with elevated KYN concentration)^44–46^. Genetical or pharmacological inhibition of KYN formation from TRP prolongs lifespan in *Drosophila*^25,43^. It was suggested that the life-extending effect of inhibition of TRP conversion into KYN depends on the decreased formation of KYN and its downstream metabolites^47^. Additionally, KYN is an immediate precursor of kynurenic acid (KYNA). Artificially increased concentration of KYNA in *Drosophila* induced a drastic increase in pupae mortality^48^. However, this does not explain the reduction in 5-HT levels in white-strain females. This could be related to a decrease in TRP availability for the 5-HT pathway, caused by tryptophan hydroxylase inhibition by high concentrations of KYN or the TRP transporter's abnormal action^49^. Females of the wild-type have a much higher baseline level of serotonin than males, so the reduction in availability or synthesis is expected to be particularly pronounced in this sex. The serotonin concentration observed in the haemolymph of black-eyed cricket imagoes remains within the range reported for mosquitoes and bedbugs, whereas there is no data on sex differences for this parameter. Single study of 5-HT levels in flies' brains indicates a lack of sex differences in this species^50^. It is known, however, that serotonin released in the hemolymph and has different functions from that in the nervous system, its concentration depends on many factors and can vary significantly^10^. There is also a scarcity of data regarding the levels of the primary neurotransmitters: dopamine and serotonin in the body of white mutant *D. melanogaster*. Single study reports a decrease in concentrations of both substances, level in the head is reduced by approximately 25%, but the analysis does not include a sex breakdown. Regrettably, no such studies using the cinnabar *Drosophila* mutant are available^51^.

Few studies involving comparisons between insects and mammals indicate a link between the TRP-KYN pathway and carbohydrate metabolism. In their study, Navrotskaya et al. used two strains of TRP-KYN pathway mutants, white and vermilion, to study the TRP-KYN pathway's relationship and response to a saccharose-rich diet in the fruit fly *D. melanogaster*^27,28^. In both cases, the tested mutant strains exhibited a different response to HSD in comparison to the control, observable as an increased tolerance to high concentrations of sucrose. In the following study, the authors focused on monosaccharides, glucose and fructose, effects: on crickets development and physiology for their immediate availability, broad usage, and direct effects on metabolism. At the same time, if with mammals and humans, the effects of glucose and fructose overconsumption are extensively studied and debated, similar studies with insects are scarce. Two primary monosaccharides were used in this study due to available literature data suggests divergent effects on *D. melanogaster.* Fructose has been shown to have a more significant potential to cause adverse effects than glucose^20^.

In the presented experiment, the house cricket, a representative of a group of insects with hemimetabolic development (Orthoptera), evolutionarily prior to holometabola such as *D. melanogaster*, was used^52,53^. The absence of a pupal stage characterises this type of development. The maturing insects display partial traits of the adult in contrast to holometabolous insects’ larvae. In which case, the larva exhibits physiology distinctly different from the adult form, with the pupal stage being the distinct transition point between these stages. The use of a hemimetabolous insect (cricket) allowed for analysis of the relationship between HMD and development with a relatively simpler, evolutionarily older insect^54^. The literature lacks information on the differences in carbohydrates metabolism between hemimetabolous and holometabolous insects and only emphasises the evolutionary conservativeness of these pathways. There are only a few studies reporting quantitative and qualitative differences between these two groups regarding metabolic processes^55^.

In the presented study, the administered HMDs had no substantial effect on crickets’ development—lack of survival decrease was observed. Only a slight but statistically significant shorter body length was observed in HMD-fed insects (Fig. 2). The larvae of the wild-type on HMD differed from the control, where in the case of the white-eyed strain, no such difference was observed. There is also a distinct difference between the strains, i.e. the white-eyed strains had a comparatively shorter body at the same time of measurement, which indicates a slower development. In *D. melanogaster* larvae developing on a medium with high levels of simple sugars, and increased developmental mortality and reduction in body size was also observed^20,21,56^.

The most substantial adverse effects of HMD on crickets are seen at the transition to the imago stage, wherein the wild-type, the imago number was decreased by half compared to the control. White-eyed crickets responded with significantly lesser changes in imago reaching success (Figs. 4 and 6). This effect is directly related to the distinctly lower sensitivity to HMD of white-eyed males in which the reduction in imago reaching success drops only from 26 to 20%, whereas for white-eyed females from 36 to 25% and wild-type males from 41 to 22%. A similar case is found regarding the time of reaching the imago, where the white strain male also exhibited the least change produced due to the monosaccharide treatment. A similar effect and strain relationship is observed in flies—HSD decreases the success of reaching the pupal stage^27,28^. In both studies, insects were fed sucrose at a concentration of 20% w/v. A comparison of Oregon (wild-type) and vermilion strains showed an increase in the time to reach the imago stage, the change in imago reaching time was approximately twice as short in vermilion as in Oregon on HSD compared to the control. A similar change in time to reach the pupal stage was observed in a second experiment with Canton S (wild-type) and white strains. In addition, the time to reach imago was assessed in this experiment with respect to sex, which did not differ in this respect^27,28^.

This indicates a role for the TRP-KYN pathway in insects' ability to manage the adverse effects of stress and high concentrations of dietary sugars. The increased resistance of crickets with elevated KYN concentrations compared to flies may indicate a lack of regulatory capacity of KYN levels alone, rather than directly its concentration in the body. An alternative explanation is a compensatory effect due to high KYN concentrations in crickets and thus an improved ability to tolerate a stressor such as HMD or other unknown mechanisms. Some studies have also demonstrated that KYNA produced from KYN may also exert beneficial, neuroprotective and antioxidant effects. It is, therefore, possible that two opposing processes are being observed: on the one hand, a negative impact on development and survival during the transformation, and on the other, reduced sensitivity to the HMD stressor^57^. The lack of data on fly mutants response to HSD or HMD with elevated KYN concentrations hinders drawing more generalised conclusions. Differences in sensitivity between males and females of the white strain crickets may also be explained by the magnitude of changes in 5-HT levels or differences in metabolic processes related, such as the accumulation of reserves for egg production in females^54^.

Juvenile forms of insects usually exhibit dynamic consumption, metabolism, and growth. They accumulate reserve materials necessary for development, especially during the moulting and during the imago stage. This is particularly important in the context of reproduction: gametes production or activity related to mate finding and copulation^58^. This allows us to hypothesise that insects have fixed physiological proportions of major biomolecules (proteins, fats, sugars) obtained due to long-term consumption of a specific diet. It can be assumed that changes in the quantity or quality of the diet will lead to changes in the proportions of biomolecules^59^. This assumption was also made in the present case, and it was chosen to investigate how HMD would affect the consumption and biochemical composition of crickets.

Comparison of the biochemical composition of cricket larvae and imago shows a relatively constant composition regarding the four types of biomolecules studied. The accumulation of reserve material mainly in the form of lipids is also evident. On the other hand, comparison of the biochemical profile of cricket and fly imagoes indicates similar body glycogen and free sugars levels. The difference is found in the amount of accumulated fat, where crickets have about five times more fat per mass unit than flies^20^. There were also no differences in the biochemical composition between crickets from the wild type and mutant strains, suggesting no mutation's impact on the underlying metabolic processes in control conditions.

Despite the evident differences in the success of imago reaching by HMD-fed crickets, no distinct disruption of biomolecule composition in the larvae's body was observed. The effect of fructose on glycogen and free monosaccharide content in wild-type strain larvae is particularly prominent in the case of HMD-fed crickets. This may indicate a different metabolism of the cricket larvae, which results in continuous depletion of ingested sugars, thus preventing the occurrence of major adverse effects of HMD. The few differences observed in white-eyed crickets' body composition indicate a potentially more substantial interaction of fructose in comparison to glucose and a lower sensitivity of the white strain to HMD when compared to the wild type. It can be suspected that the regulatory mechanisms themselves, e.g., insulin-like peptides (ILPs) during the development, are predominantly affected, which impacts the success of reaching the imago^60^. There is also a known link between carbohydrate regulatory mechanisms and key hormones that regulate insect growth and development (ecdysone, juvenile hormone). It is likely that high dietary sugar levels, leading to elevated ILPs activity, result in disrupted hormone secretion and observed imago reaching success^61^.

When compared to larvae, changes in the biochemical composition of imago reach a comparable scale. Still, similarly to the larvae, there are no changes in the quantity of protein, but there are variations in the quantity of stored fat—the primary reserve material. These alterations are observed in both sexes of the wild strain and the white-eyed strain fed with a high-fructose diet. A reduced glycogen level is also apparent in the white-eyed strain females compared to the fructose-fed black-eyed females. After reaching the imago, fructose-fed wild-type insects had elevated free sugars levels, whereas no similar effect was observed in the white strain crickets. These results confirm the lower sensitivity of the white strain to high concentrations of monosaccharides in the diet and the overall more pronounced effects of fructose on cricket physiology and metabolism.

When fruit flies are exposed to media containing high sugar concentrations, they experience extensive biochemical and physiological changes. In *D. melanogaster* imago developing on a medium with high levels of simple sugars (concentration 20% w/v), an increase of lipids and glycogen concentration is observed^20,21^. It is also reported that males and females react differently, with males reacting more acutely at the level of enzymatic activity (metabolic and antioxidant enzymes) at 10% HMD^56^. Moreover, there is a lack of data on the protein content of the body of the flies studied. Also, no information is available regarding body composition changes in TRP-KYN mutants flies.

It has been suggested that the observed differences in the effects of fructose and glucose may be due to the ability of fructose to engage in a number of catalytic reactions other than glycolysis, sometimes called fructolysis and initiated by fructokinase (ketohexokinase). This can lead to a couple of important effects. Firstly, the product of the fructokinase reaction, fructose-1-phosphate, has regulatory functions, e.g. inhibiting glycogenolysis. Additionally, it can activate glycogen synthase. Both mechanisms explain why increased fructose intake can lead to the glycogen accumulation observed in some experiments. The second mechanism involves catalysis where fructose-1-phosphate undergoes catalytic hydrolysis with the formation of dihydroxyacetone phosphate (DHAP) and glyceraldehyde. DHAP is a precursor to lipid biosynthesis, it is its incorporation into lipids, especially triacylglycerols, that requires fewer steps than for glycolysis starting with glucose^20^. For the biochemical effects observed in crickets for the HMDs used where greater changes are seen in lipid rather than glycogen levels the second mechanism seems to fit better.

Analysis of food consumption levels indicated a reduction in consumption in the HMD-treated groups. The differences are most likely a consequence of regulatory mechanisms based on the association between food, consumption, and growth^62^. Interesting is the difference between consumption calculated per individual or mass increase. In the first case, both HMS wild strain groups had lower consumption values than control, wherein white strain only glucose-rich diet consumption values were lower. When consumption was calculated in relation to growth, the relation between wild type groups was inverted, and white strain crickets had lower consumption only in the glucose group. This observation confirms that in the case of energetically crucial substances, it is important to monitor consumption more closely. This shows that the insects treated with HMD consumed significantly less, achieving the same mass gain. In the wild-type, both HMDs reduced food consumption, while in the white-eyed strain, only a glucose-rich diet induced a significantly lower effect (Fig. 2e,f). The differences observed in food consumption could be attributed to sensory information on the "sweetness" of the ingested food and the bioavailability of sugars in the diet; despite similar energy values of the nutrient solutions used (Supplementary Table S1), flies showed a reduction in food consumption when fed high sugar diets, indicating a possible corrective mechanism regulating the amount of food intake depending on its calorific value^63^. The lack of differences in frass caloric content indicates an energy redundancy of the food in relation to the organism's metabolic needs. As such, this indicates a direct effect of the tested sugars instead of the caloricity of the food on the observed results, which is also confirmed by the caloric analysis of the diet.

It is also worth noting the lack of change in water content in the body of crickets, which is, however, observed in flies, where the water content dropped to about 40% for a diet with 20% monosaccharides^20^. This phenomenon can be explained by the loss of metabolic water. However, it may be due to the specificity of the food these two insects consume. Crickets are provided with a separate source of water ad libitum. The flies, however, ingest the water together with the feed, forcing them to consume the sugars it contains continually. In addition, increasing sugar concentration in the nutrient solution proportionally reduces the water content. A single paper reports that observed disturbances in flies resulting from an HSD diet may result from impaired water balance rather than the direct effect of sugars^64^.

The presented results are the first to address the effects of an HMD on a hemimetabolous insect's development and biochemistry. In addition, this research involved the mutants of *A. domesticus* with altered TRP-KYN pathway—the white eye (white) strain. The results indicate an adverse effect of a monosaccharide-rich diet on cricket development's rate and imago reaching success. At the same time, the white mutant strain crickets (especially males), with impaired TRP metabolism, were less sensitive to HMD, indicating the apparent differences in the response of the hemi- and holometabolous insect to HMD. In comparison to *A. domesticus, D. melanogaster* exposed to HMD expressed a greater extent of body mass changes, water content, and biomolecule composition. However, some similarities between holo- and hemimetabolous models can also be observed, especially in relation to development, the more potent effects of fructose, and the similar role of the TRP-KYN pathway in response to HMD. The observed lesser differences between mutant and wild-type crickets in response to HMD compared to studies involving *D. melanogaster* mutants might result from higher rather than lower concentrations of KYN in cricket haemolymph or from a different effect of sucrose on insects' physiology. The lack of distinct effects of HMD on survival and biochemical profile of cricket larvae as compared to fly larvae may indicate a different response to HMD and thus differences in regulatory processes at the level of carbohydrate homeostasis. Thus, indicating large differences between these insects in an evolutionary and metabolic context^55^. The presented conclusions provide a basis for further studies on the insects' metabolism involving sucrose administration as well as experimental setups employing a broader spectrum of physiological parameters.

## Materials and methods

### Animal culture

In conducted experiments, insects reared in the Institute of Biology, Biotechnology and Environmental Protection (Faculty of Natural Sciences, the University of Silesia in Katowice), from wild-type (black eye) and mutated strain (white eye) were used. Culture conditions were kept stable at 30 °C, 40% RH, and light regime 12:12 LD. As a parental group, four sets of 10 females and males of similar age from each strain were chosen. Ten days after hatching, the larvae were placed in experimental boxes (50 larvae per box). In all of the developmental assays, four boxes were prepared for each of the concentrations. A similar setup was prepared for all of the tests.

In described experiments, the crickets were fed with KanisanQ rodent pellets (Sano, dry mass 88%, 16.5% crude protein, 3.7% lipids, 9% crude fiber, 6.5% crude ash, 61.3% carbohydrates; main ingredients: dried grass, wheat, barley, soy, corn, sprouts, vegetable oil, calcium carbonate, sodium chloride, monocalcium phosphate) used in the continuous rearing of crickets. The HMD was prepared by adding glucose and fructose (Sigma, 99%) to pulverised pellets, thoroughly mixing the resulting mass with water and subsequent drying. The control diet was prepared likewise but without added sugars.

### Hemolymph concentration of KYN/TRP

10-day-old imagos of both sexes from both strains were isolated on the day of the imaginal moult and placed in separate containers. After ten days, the hemolymph was collected. Five microliters of hemolymph were collected from the clipped leg of the 3rd pair and transferred to 10 µL of ice-cold PBS. A single sample contained hemolymph from three individuals (final volume 45 µL), and the number of replicates per group equalled 10. TRP and KYN content was measured using an ELISA assay performed using a commercially available kit (Kynurenine/Tryptophan ratio ELISA pack, ImmuSmol). The quantification was conducted with a Tecan M200 spectrophotometer.

### Hemolymph concentration of serotonin

The serotonin level assay was performed as same as the determination of TRP and KYN content described above. However, the single sample consisted of the hemolymph from five individuals in PBS (final volume 50 µL). The number of replicates per group equalled 10. Serotonin (5-HT) content was determined using the ELISA method conducted with a commercial kit (Serotonin Research ELISA ImmuSmol). The quantification was performed with a Tecan M200 spectrophotometer.

### HSD treatments

A primary developmental experiment was divided into two parts. During the first one, only wild-type (black-eyed strain) crickets were used. Ten days old larvae were divided into groups, 50 individuals per box. For every experimental group, three boxes with larvae were used. In this part, glucose and fructose were used in three concentrations: 10, 15, and 20% w/v. At this point, identification of effective concentration of sugars in food on crickets development was assessed. Food and water were available ad libitum. The percentage of insects reaching imago was quantified by counting appearing imago every 2 days.

In the second part of the experiments, only one sugar concentration was used. The 20% concentration of monosaccharides was based on the available literature data and conducted pilot studies (Supplementary Fig. S1). HMD with 20% of additional glucose or fructose in food was prepared. Ten days old larvae from both strains, wild-type, and white, were used. On 30th and 40th day, boxes with crickets were photographed for subsequent survival and body length calculation (average values for boxes were used for further statistical analysis). Body length was calculated for 45 individuals per box with the use of ImageJ^65^. The percentage of insects reaching imago was analysed by counting emerging imagos every two days. Imagos have been weighed 24 h after moulting and freezed (− 70 °C) for further analysis.

### Calorimetry measurements

Measurements of caloric values of prepared food and collected insects’ frass were conducted. Frass was collected from boxes with larvae on the 30th day of the experiment. On the 25th day of the experiment, boxes were cleaned to collect fresh frass from a 5-day-long period. Every type of food (control and HMD diet) and frass powdered and subsequently compressed into tablets for calorimetric assay. The average sample mass equalled 60 mg, four samples per group was measured. The measurements were carried out using the Parr 6772 Calorimetric Thermometer system. Caloricity of prepared food was established (Supplementary Table S1).

### Food consumption test

For consumption assay, new sets of boxes with larvae were prepared as same as previously described—10 days old larvae (both strains) 50 per box (6 boxes) like in the primary experiment. On the 30th day of larvae life, ten individuals were weighed and transferred to smaller boxes (dimensions) with cover, water, and a known amount of experimental food—six replications per group. After 5 days, uneaten food was collected from boxes and weighed. Alive individuals in boxes were counted and weighed (no mortality was observed). Baseline consumption levels were calculated as the amount of food (mg) consumed per insect. Final consumption was calculated as an amount of consumed food to insects biomass increase (mg/∆100 mg).

### Biochemical profile

#### Tissues preparation

Whole insects (larvae and imago) were sacrificed for biochemical composition quantification (n = 6). All four groups of biomolecules (proteins, fats, sugars—glycogen, and free sugars) were assessed for each insect^66^. This approach allowed to observe relational changes in body composition. Crickets were homogenised in the ALB buffer. For every 15 mg of fresh body mass, 180 µL aqueous lysis buffer was used. Homogenate was centrifuged for 5 min at 4 °C, thereafter 5 µL of supernatant was collected for protein content analysis. Subsequently, 180 µL of supernatant was used for further analysis, 20 µL of 20% Na_2_SO_4_ solution was added, mixed, and 1500 µL of chloroform: methanol (1:2) was added and mixed again. Solutions were centrifuged at 180*g* for 15 min in 4 °C. The obtained supernatant was used to quantify carbohydrates and lipids content and the pellet for glycogen content.

#### Proteins

Protein content was quantified using the Bradford method^67^. 5 µL of sample from the homogenate was transferred to the well of 96 wells Corning plate, and 150 µL of Bradford reagent was added. The solution was incubated in the dark for 10 min, and then the absorbance was measured at 595 nm using a Tecan M200 reader. Protein content was calculated using the albumin calibration curve.

#### Free carbohydrates

To measure free carbohydrates, 150 µL of supernatant was transferred to an Eppendorf tube placed in a heat block set to 50 °C for 30 min in order to concentrate the solution down to 30 µL. Subsequently, 240 µL of anthrone reagent was added to each sample. In the next step, samples were incubated for 15 min at 90 °C, and 200 µL of each sample was transferred to the 96 wells Corning plate. Carbohydrate concentration was measured using a plate reader (Tecan M200) at 625 nm wavelength. Each sample was read three times as a technical repetition. The mixture of methanol and chloroform with an anthrone reagent was used as a blind read. Carbohydrates concentration was calculated from the glucose calibration curve.

#### Lipids

To measure lipids, 100 µL of previously prepared supernatant was collected and incubated at 90 °C until complete evaporation. In the next step, 10 µL of H_2_SO_4_ (min. 95%) was added, and the samples were incubated at 90 °C for 2 min. After cooling on ice, 190 µL of vanillin reagent was added. Samples were mixed and incubated at room temperature for 15 min. The amount of lipids were measured in a plate reader (Tecan M200) using 96 wells Corning plate in 525 nm. Every sample was read three times as technical repetitions. The mixture of methanol and chloroform with vanillin reagent was used as a blind read. Lipids concentration was calculated from the triolein calibration curve.

#### Glycogen

Pellet left after supernatant removal was resuspended in 400 µL of 80% methanol and centrifuged for 5 min, 16,000*g* in 4 °C. The procedure was repeated twice. The supernatant was discarded, and 1 mL of anthrone reagent was added. Thereafter, samples were incubated for 15 min at 90 °C. In the next step, samples were placed on ice to halt the reaction. The mixture in the volume of 200 µL was transferred to 96 wells Corning plate and read with Tecan M200 in wavelength 625 nm. For the calculation of the glycogen concentration, a glucose calibration curve was used. As a blind read, an anthrone reagent in ABL was used.

### Imago body mass and water content

Imago (24 h after moulting) of both sex from every group were collected. Insects were weighed and stored at − 70 °C for 24 h. After that period, insects were freeze-dried (Christ Alpha 1–4) for 24 h. The dry mass of six crickets of both sex from every group was determined, and the percentage of water content was calculated. For body mass analysis, data from observations of imago reaching were used (minimum n = 10) (4.4).

### Statistical analyses

For basic between-group comparisons, parametric tests for analysis of variance (ANOVA) were used. Toanalysee the homogeneity of distributions, the Brown–Forsythe ANOVA test (P < 0.05) was used. For multiple comparisons, Tukey's multiple comparisons ANOVA test (P < 0.05) was employed, and for data exhibiting inhomogeneity of standard distributions—Dunnett's T3 multiple comparisons test (P < 0.05). Analyses and graphs were obtained using GraphPad Prism software (version 9).

#### Effect of sugars on levels of biomolecules

To assess monosaccharide treatment’s effect on body mass and the level of measured biomolecules, linear regression models were fitted independently for body mass and each biomolecule. During analyses, data from cricket larvae and adults were handled separately. In the larvae, the response variable was either body mass, level of lipids, proteins, glycogen, or glucose, while predictor variables were treatment group (control, fructose or glucose), phenotype (wild or mutant), and the interaction between treatment group and phenotype. The models of adult crickets were similar, with the difference that we included sex and its interaction with treatment and phenotype as predictors. In analyses of both larvae and adults, the statistical significance of interaction terms was tested using variance tables analysis, and when an interaction term was not significant, reduced models were fitted, omitting the non-significant interaction term.

#### Effect of sugars on maturing success

To test how sugar treatments affected maturing success Cox proportional hazards regression model was fitted using the R-package “survival”^68,69^. The model predictors were: treatment group, phenotype, sex, and interactions between the three predictors.

## Conclusions

1. A diet rich in monosaccharides at concentrations greater than 10% significantly reduced the success of imago reaching in *A. domesticus* crickets.
2. Fructose had a more substantial effect and generated greater differences between the test groups than glucose.
3. The white-eyed strain, with impaired tryptophan metabolism, displayed greater tolerance to high concentrations of monosaccharides and therefore showed less severe adverse effects of HMD.
4. Furthermore, sex moderated changes in imago reaching success and biocompunds profile, with white-eyed males showing the least sensitivity to the disruptive effects of both fructose and glucose.

## Supplementary Information

Supplementary Information.

## References

[1] Gostner JM (2020). Tryptophan metabolism and related pathways in psychoneuroimmunology: The impact of nutrition and lifestyle. Neuropsychobiology.

[2] Peters, J. C. Tryptophan nutrition and metabolism: an overview. In *Advances in Experimental Medicine and Biology* vol. 294 345–358 (Springer, 1991). 10.1007/978-1-4684-5952-4_32 1772073

[3] Yao K (2011). Tryptophan metabolism in animals: Important roles in nutrition and health. Front. Biosci. Sch..

[4] Gao J (2018). Impact of the gut microbiota on intestinal immunity mediated by tryptophan metabolism. Front. Cell. Infect. Microbiol..

[5] Badawy, A. A.-B. Tryptophan metabolism: A versatile area providing multiple targets for pharmacological intervention. *Egypt. J. Basic Clin. Pharmacol.* **9**, (2019). 10.32527/2019/101415 PMC6520243 31105983

[6] Grubbs N, Haas S, Beeman RW, Lorenzen MD (2015). The ABCs of eye color in *Tribolium castaneum*: Orthologs of the Drosophila white, scarlet, and brown Genes. Genetics.

[7] White LD, Coates CJ, Atkinson PW, O’Brochta DA (1996). An eye color gene for the detection of transgenic non-drosophilid insects. Insect Biochem. Mol. Biol..

[8] Allegri G, Costa CVL, Bertazzo A, Biasiolo M, Ragazzi E (2003). Enzyme activities of tryptophan metabolism along the kynurenine pathway in various species of animals. Farmaco.

[9] van der Goot AT, Nollen EAA (2013). Tryptophan metabolism: Entering the field of aging and age-related pathologies. Trends Mol. Med..

[10] Bacqué-cazenave J (2020). Serotonin in animal cognition and behavior. Int. J. Mol. Sci..

[11] Li Y, Hu N, Yang D, Oxenkrug G, Yang Q (2017). Regulating the balance between the kynurenine and serotonin pathways of tryptophan metabolism. FEBS J..

[12] Oxenkrug GF (2010). The extended life span of *Drosophila melanogaster* eye-color (white and vermilion) mutants with impaired formation of kynurenine. J. Neural Transm..

[13] Cervenka, I., Agudelo, L. Z. & Ruas, J. L. Kynurenines: Tryptophan’s metabolites in exercise, inflammation, and mental health. *Science* **357** (2017). 10.1126/science.aaf9794 28751584

[14] Badawy, A. A. Tryptophan metabolism : A versatile area providing multiple targets for pharmacological intervention. **9**, (2019). 10.32527/2019/101415 PMC6520243 31105983

[15] Oxenkrug GF, Turski WA, Zgrajka W, Weinstock JV, Summergrad P (2013). Tryptophan-kynurenine metabolism and insulin resistance in hepatitis C patients. Hepat. Res. Treat..

[16] Rebnord EW (2017). The kynurenine:tryptophan ratio as a predictor of incident type 2 diabetes mellitus in individuals with coronary artery disease. Diabetologia.

[17] Oxenkrug GF (2015). Increased plasma levels of xanthurenic and kynurenic acids in type 2 diabetes. Mol. Neurobiol..

[18] Shamim GG, Ranjan SK, Pandey DM, Ramani R (2014). Biochemistry and biosynthesis of insect pigments. Eur. J. Entomol..

[19] Pandey UB, Nichols CD (2011). Human disease models in *Drosophila melanogaster* and the role of the fly in therapeutic drug discovery. Pharmacol. Rev..

[20] Rovenko BM (2015). High consumption of fructose rather than glucose promotes a diet-induced obese phenotype in *Drosophila melanogaster*. Comp. Biochem. Physiol. Part A Mol. Integr. Physiol..

[21] Musselman LP (2011). A high-sugar diet produces obesity and insulin resistance in wild-type Drosophila. DMM Dis. Model. Mech..

[22] Ferre, J., Silva, F., Real, M. D. & Mensua, J. L. Comparative study of the eye colour mutants of *Drosophila melanogaster*: Quantification of the eye-pigments and related metabolites. In *Chemistry and Biology of Pteridines. Pteridines and Folic Acid Derivatives* (ed. Blair, J.) 669–674 (De Gruyter, 1983). 10.1515/9783111619453.669.

[23] Mackenzie SM (1999). Mutations in the white gene of *Drosophila melanogaster* affecting ABC transporters that determine eye colouration. Biochim. Biophys. Acta Biomembr..

[24] Mackenzie SM, Howells AJ, Cox GB, Ewart GD (2000). Sub-cellular localisation of the white/scarlet ABC transporter to pigment granule membranes within the compound eye of *Drosophila melanogaster*. Genetica.

[25] Navrotskaya V, Wnorowski A, Turski W, Oxenkrug G (2018). Effect of kynurenic acid on pupae viability of *Drosophila melanogaster* cinnabar and cardinal eye color mutants with altered tryptophan-kynurenine metabolism. Neurotox. Res..

[26] Kashio S, Miura M (2020). Kynurenine metabolism in the fat body non-autonomously regulates imaginal disc repair in Drosophila. iScience.

[27] Navrotskaya V, Oxenkrug G, Vorobyova L, Summergrad P (2016). Attenuation of high sucrose diet-induced insulin resistance in ABC transporter deficient white mutant of *Drosophila melanogaster*. Integr. Obes. Diabetes.

[28] Navrotskaya V, Oxenkrug G, Vorobyova L, Summergrad P (2015). Attenuation of high sucrose diet-induced insulin resistance in tryptophan 2,3-dioxygenase deficient *Drosophila melanogaster* vermilion mutants. Integr. Obes. Diabetes.

[29] Francikowski J (2019). Characterisation of white and yellow eye colour mutant strains of house cricket, *Acheta domesticus*. PLoS One.

[30] Bawa M, Songsermpong S, Kaewtapee C, Chanput W (2020). Effect of diet on the growth performance, feed conversion, and nutrient content of the house cricket. J. Insect Sci..

[31] Badisco L (2008). Purification and characterization of an insulin-related peptide in the desert locust, *Schistocerca gregaria*: Immunolocalization, cDNA cloning, transcript profiling and interaction with neuroparsin. J. Mol. Endocrinol..

[32] Kushida A (2012). Xanthurenic acid is an endogenous substrate for the silkworm cytosolic sulfotransferase, bmST1. J. Insect Physiol..

[33] Stratakis E (1980). Tryptophan metabolism during development of the stick insect, *Carausius morosus* Br. tissue distribution and interrelationships of metabolites of the kynurenine pathway. J. Comp. Physiol..

[34] Rizki T, Rizki R (1964). Factors affecting the intracellular synthesis of kynurenine. J. Cell Biol..

[35] Brown TM (2001). A single gene (yes) controls pigmentation of eyes and scales in *Heliothis virescens*. J. Insect Sci..

[36] Dustmann JH (1987). Eye-colour mutants of the honeybee. Bee World.

[37] Snodgrass GL (2002). Characteristics of a red-eye mutant of the tarnished plant bug (Heteroptera: Miridae). Ann. Entomol. Soc. Am..

[38] Liu S-H (2014). Biological and biochemical characterization of a red-eye mutant in *Nilaparvata lugens* (Hemiptera: Delphacidae). Insect Sci..

[39] Oxenkrug, G. Insulin resistance and dysregulation of tryptophan-kynurenine and kynurenine-nicotinamide adenine dinucleotide metabolic pathways. In *Molecular Neurobiology* vol. 48 294–301 (Humana Press Inc., 2013). 10.1007/s12035-013-8497-4 PMC3779535 23813101

[40] Figon F, Casas J (2019). Ommochromes in invertebrates: Biochemistry and cell biology. Biol. Rev..

[41] Ferre J (1983). Accumulation of kynurenic acid in the “cinnabar” mutant of *Drosophila melanogaster* as revealed by thin-layer chromatography. Insect Biochem. (Insect Biochem.).

[42] Cerstiaens A (2003). Neurotoxic and neurobehavioral effects of kynurenines in adult insects. Biochem. Biophys. Res. Commun..

[43] Oxenkrug G, Navrotskaya V, Vorobyova L, Summergrad P (2011). Extension of life span of drosophila Melanogaster by the inhibitors of tryptophan-kynurenine metabolism. Fly (Austin)..

[44] Zakharov GA, Zhuravlev AV, Payalina TL, Kamyshev NG, Savvateeva-Popova EV (2011). The influence of *D. melanogaster* mutations of the kynurenine pathway of tryptophan metabolism on locomotor behavior and expression of genes belonging to glutamatergic and cholinergic systems. Ecol. Genet..

[45] Savvateeva-Popova, E. V., Popov, A. V., Heinemann, T. & Riederer, P. Drosophila mutants of the kynurenine pathway as a model for ageing studies. In *Advances in Experimental Medicine and Biology* vol. 527 713–722 (Kluwer Academic/Plenum Publishers, 2003). 10.1007/978-1-4615-0135-0_84 15206794

[46] Savvateeva E (2000). Age-dependent memory loss, synaptic pathology and altered brain plasticity in the Drosophila mutant cardinal accumulating 3-hydroxykynurenine. J. Neural Transm..

[47] Oxenkrug GF (2007). Genetic and hormonal regulation of tryptophan kynurenine metabolism: Implications for vascular cognitive impairment, major depressive disorder, and aging. Ann. N. Y. Acad. Sci..

[48] Navrotskaya, V. & Oxenkrug, G. Effect of kynurenic acid on development and aging in wild type and vermilion mutants of *Drosophila melanogaster*. *Pharmacol. Drug Dev. Ther.* **1**, (2016). 10.15761/PDDT.1000104 PMC5381657 28393115

[49] Vleugels R, Verlinden H, Broeck JV (2015). Serotonin, serotonin receptors and their actions in insects. Neurotransmitter.

[50] Denno ME, Privman E, Venton BJ (2014). Analysis of neurotransmitter tissue content of *Drosophila melanogaster* in different life stages. ACS Chem. Neurosci..

[51] Borycz JA (2008). Drosophila ABC transporter mutants white, brown and scarlet have altered contents and distribution of biogenic amines in the brain. J. Exp. Biol..

[52] Jindra M (2019). Where did the pupa come from? The timing of juvenile hormone signalling supports homology between stages of hemimetabolous and holometabolous insects. Philos. Trans. R. Soc. B Biol. Sci..

[53] Truman JW (2019). The evolution of insect metamorphosis. Curr. Biol..

[54] Horch, W. H., Mito, T., Popadić, A., Ohuchi, H. & Noji, S. *The Cricket as a Model Organism: Development, Regeneration, and Behavior* (Springer Japan, 2017). 10.1007/978-4-431-56478-2.

[55] Bernays EA (1986). Evolutionary contrasts in insects: Nutritional advantages of holometabolous development. Physiol. Entomol..

[56] Lushchak OV, Rovenko BM, Gospodaryov DV, Lushchak VI (2011). Drosophila melanogaster larvae fed by glucose and fructose demonstrate difference in oxidative stress markers and antioxidant enzymes of adult flies. Comp. Biochem. Physiol. A Mol. Integr. Physiol..

[57] Campesan S (2011). The kynurenine pathway modulates neurodegeneration in a Drosophila model of Huntington’s disease. Curr. Biol..

[58] Smykal V, Raikhel AS (2015). Nutritional control of insect reproduction. Curr. Opin. Insect Sci..

[59] van Schoor T, Kelly ET, Tam N, Attardo GM (2020). Impacts of dietary nutritional composition on larval development and adult body composition in the yellow fever mosquito (*Aedes aegypti*). Insects.

[60] Wu Q, Brown MR (2006). Signaling and function of insulin-like peptides in insects. Annu. Rev. Entomol..

[61] Murillo-Maldonado JM, Sánchez-Chávez G, Salgado LM, Salceda R, Riesgo-Escovar JR (2011). Drosophila insulin pathway mutants affect visual physiology and brain function besides growth, lipid, and carbohydrate metabolism. Diabetes.

[62] Lushchak OV (2014). Specific dietary carbohydrates differentially influence the life span and fecundity of *Drosophila melanogaster*. J. Gerontol. Ser. A Biol. Sci. Med. Sci..

[63] Semaniuk U (2018). Within-diet variation in rates of macronutrient consumption and reproduction does not accompany changes in lifespan in *Drosophila melanogaster*. Entomol. Exp. Appl..

[64] van Dam E (2020). Sugar-induced obesity and insulin resistance are uncoupled from shortened survival in Drosophila. Cell Metab..

[65] Schneider CA, Rasband WS, Eliceiri KW (2012). NIH Image to ImageJ: 25 years of image analysis. Nat. Methods.

[66] Foray V (2012). A handbook for uncovering the complete energetic budget in insects: the van Handel’s method (1985) revisited. Physiol. Entomol..

[67] Bradford MM (1976). A rapid and sensitive method for the quantitation of microgram quantities of protein utilizing the principle of protein-dye binding. Anal. Biochem..

[68] Therneau, T. M. & Grambsch, P. M. *Modeling Survival Data: Extending the Cox Model*. (Springer, 2000). 10.1007/978-1-4757-3294-8.

[69] R: a language and environment for statistical computing. https://www.gbif.org/tool/81287/r-a-language-and-environment-for-statistical-computing.

