# Supplementary material for: The effects of high-monosaccharide diets on development and biochemical composition of white-eyed mutant strain of house cricket (Acheta domesticus)
Source: Sci Rep. 2021 Oct 27;11:21147. doi: 10.1038/s41598-021-00393-5 (PMC8551166; doi:10.1038/s41598-021-00393-5)
Supplement: Supplementary file 1 — Supplementary Information. [file 41598_2021_393_MOESM1_ESM.docx]

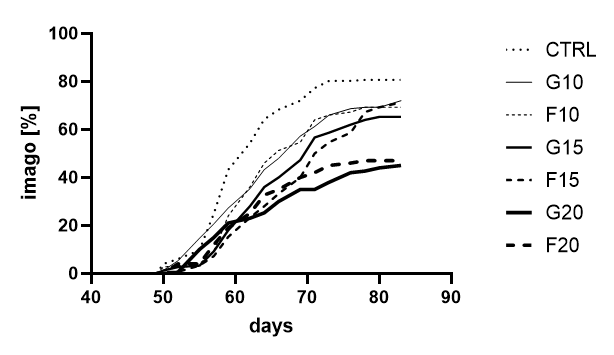


Figure S1. Black eyed strain success of imago reaching for pilot research with three different concentrations of monosaccharides in diet (10, 15, 20% of glucose (G) and fructose (F)).

Table S1.The caloricity of the control feed and of the HMDs

| **Diet** | **Average caloricity [J/g dry mass]** | **±SD** |
| --- | --- | --- |
| food with 20% glucose | 17152.1 | 39.4 |
| food with 20% fructose | 17167.5 | 107.7 |
| control food | 17692.5 | 177.5 |
